# Geographic and Sociodemographic Factors and Receipt of Metabolic Disease Specialty Care

**DOI:** 10.1001/jamanetworkopen.2025.11559

**Published:** 2025-05-20

**Authors:** Margaret F. Zupa, Scott D. Rothenberger, Jessica G. Bauer, Yihao Zheng, Amber E. Johnson, Ellen Kinnee, Ann-Marie M. Rosland

**Affiliations:** 1 Division of Endocrinology and Metabolism, University of Pittsburgh School of Medicine, Pittsburgh, Pennsylvania; 2 Caring for Complex Chronic Conditions Research Center, University of Pittsburgh, Pittsburgh, Pennsylvania; 3 Center for Research on Healthcare Data Center, University of Pittsburgh, Pittsburgh, Pennsylvania; 4 Division of General Internal Medicine, University of Pittsburgh School of Medicine, Pittsburgh, Pennsylvania; 5 Section of Cardiology, Department of Medicine, University of Chicago, Chicago, Illinois; 6 University Center for Social and Urban Research, University of Pittsburgh, Pittsburgh, Pennsylvania; 7 Center for Health Equity Research and Promotion, VA Pittsburgh Health System, Pittsburgh, Pennsylvania

## Abstract

**Importance:**

Atherosclerotic cardiovascular disease is the leading cause of death among adults with type 2 diabetes in the US. Endocrinology and cardiology care may improve outcomes for these patients, but access to this care is limited for many patients by practitioner shortages in rural areas and other barriers.

**Objective:**

To assess associations of geographic and sociodemographic factors with endocrinology and cardiology care receipt among adults with type 2 diabetes and atherosclerotic cardiovascular disease before and after widespread telemedicine uptake.

**Design, Setting, and Participants:**

This retrospective cohort study used electronic medical record and public geographic and infrastructure data at a large health system spanning urban and rural counties in Pennsylvania. Participants were adults with type 2 diabetes and atherosclerotic cardiovascular disease who received primary care from January 2018 to June 2022.

**Exposures:**

Patient-level geographic measures, including distance to clinic, public transit, and cellular data access, and sociodemographic factors, including age, gender, race, and neighborhood socioeconomic status.

**Main Outcomes and Measures:**

The primary outcome was at least 1 outpatient visit with endocrinology or cardiology. Geospatial analysis assessed patient-level geographic factors using zip code centroids, and separate multivariable logistic regression models evaluated associations between variables and endocrinology and cardiology care receipt. Analyses were stratified by period before (January 1, 2018, to March 15, 2020) and after (March 16, 2020, to June 30, 2022) COVID-19 pandemic–related telemedicine uptake; mixed-effects models tested for differences between periods.

**Results:**

Of 9546 adults (mean [SD] age, 68.5 [10.0] years; 5854 male [61%]; 82 Asian [1%]; 930 Black [10%]; 8451 White [89%]; 7877 urban [83%]), 1747 received endocrinology care and 5578 received cardiology care. In the pretelemedicine period, distance to endocrinology clinic (adjusted odds ratio [aOR] per 10 miles, 0.74; 95% CI, 0.64-0.84) and older age (aOR per 10 years, 0.70; 95% CI, 0.66-0.75) were associated with lower odds of receiving endocrinology care. In the posttelemedicine period, the aOR for distance to clinic increased, but that for older age decreased. Black patients were less likely than White patients to receive cardiology care in the pretelemedicine period (aOR, 0.71; 95% CI, 0.60-0.82), and this association persisted in the posttelemedicine period.

**Conclusions and Relevance:**

In this cohort study of adults with type 2 diabetes and atherosclerotic cardiovascular disease, geographic and sociodemographic factors were associated with receipt of endocrinology and cardiology care. Widespread availability of telemedicine may enhance equitable access to endocrinology care for patients facing geographic barriers, but disparities in use of specialty care by age and race persisted in the posttelemedicine period.

## Key Points

**Question:**

Are geographic and sociodemographic factors that may impact access to care, including telemedicine, associated with receipt of metabolic disease specialty care?

**Findings:**

In this cohort study of 9546 adults with type 2 diabetes and atherosclerotic cardiovascular disease, greater distance to clinic and older age were associated with lower odds of receiving endocrinology care. Black adults were less likely than White adults to receive cardiology care.

**Meaning:**

These findings suggest that strategies to overcome identified barriers could increase equitable access to metabolic disease specialty care for adults with type 2 diabetes and atherosclerotic cardiovascular disease.

## Teaser

This cohort study examines associations of geographic and sociodemographic factors with endocrinology and cardiology care receipt among adults with type 2 diabetes and atherosclerotic cardiovascular disease, before and after widespread telemedicine uptake.

## Introduction

Among adults with type 2 diabetes (T2D) in the US, atherosclerotic cardiovascular disease (ASCVD) is the leading cause of death.^1^ Treatment guidelines promote blood glucose and blood pressure goals and the use of specific medications to reduce cardiovascular risk for these patients.^2,3^ However, the proportion of American adults with T2D meeting these metrics has declined since 2010.^4^ In addition, disparities in outcomes (including hemoglobin A_1c_, diabetes-related complications, guideline-concordant medication use, and mortality) by race and ethnicity,^5,6,7^ socioeconomic status,^8,9^ and rurality^10,11,12,13^ are well established. Care from specialists in metabolic disease, specifically endocrinologists and cardiologists, in conjunction with primary care practitioners, has been associated with increased use of guideline-concordant medications and achievement of treatment goals for adults with T2D,^14,15^ high cardiovascular risk,^16^ and coronary artery disease.^17^ Thus, appropriate delivery of metabolic disease specialty care may be one approach to improve outcomes for adults with T2D and ASCVD, especially among populations who experience worse clinical outcomes.

However, many patients face barriers to accessing metabolic disease specialty care. With concentration of endocrinologists and cardiologists in urban centers, patients in rural communities face geographic barriers to care.^18,19^ Other barriers, including lack of transportation, may predominantly affect underresourced populations.^20^ Ideally, receipt of specialty care for T2D and ASCVD would be determined by clinical factors, such as disease complexity and comorbid conditions, rather than nonclinical factors, such as rurality, race, ethnicity, and socioeconomic status. However, these nonclinical factors, which are linked to disparities in clinical outcomes, may also impact access to and use of this care. To our knowledge, the association of patient geographic and sociodemographic characteristics with metabolic disease specialty care receipt has not previously been assessed. In this study, we aimed to determine the association of these factors with receipt of endocrinology and cardiology care among adults with T2D and ASCVD to gain insight into the association of these nonclinical factors with access to this care. Because telemedicine may facilitate access for patients with barriers to in-person care, we also assessed the association of widespread telemedicine uptake in 2020 with specialty care receipt.

## Methods

### Study Design, Cohort, and Setting

We performed a retrospective cohort study using geospatial analysis to link patient-level medical record data with public geographic and infrastructure data. Adults with T2D and ASCVD seen within a single large health system between 2018 to 2022 were included in the study cohort. Patients had to be aged 18 to 90 years, with a diagnosis code for T2D and ASCVD documented at least twice in outpatient encounters in the 2 years preceding the observation period (2016-2018), and have more than 3 primary care visits: 1 between 2016 and 2018, 1 in the pretelemedicine period, and 1 in the posttelemedicine period. We defined the pretelemedicine period as January 1, 2018, to March 15, 2020, and the posttelemedicine period as March 16, 2020, to June 30, 2022; patients were followed up through both periods from 2018 to 2022. Finally, patients had to have a residential address in Pennsylvania. This study was determined to be exempt from informed consent by the University of Pittsburgh institutional review board because the data were deidentified, in accordance with 45 CFR §46. The results are reported in accordance with Strengthening the Reporting of Observational Studies in Epidemiology (STROBE) reporting guidelines.^21^

The health system in which this study was conducted includes over 800 outpatient offices and 40 hospitals in urban and rural counties in western Pennsylvania. After broadened coverage by the Centers for Medicare & Medicaid Services and other insurers in early 2020, there was rapid expansion of telemedicine use, with more than 6000 telemedicine visits conducted per day in this health system early in the COVID-19 pandemic.^22^ Nearly 1 in 3 adults with T2D receiving endocrinology care in this system from May 2020 to May 2022 used exclusively telemedicine.^23^ Cardiology also had substantially increased use of telemedicine, although uptake was lower than in endocrinology, consistent with national trends.^24^

### Geographic Measures

Patients were mapped to their population-weighted zip code centroids using their 5-digit zip code. Population-weighted zip code centroids assign patients to the geographic area of their home zip code where the most people reside. Specialty clinic addresses within Pennsylvania were obtained from the National Provider Identifier Registry and were augmented by the local health system directory. Drive distance in miles and time in minutes to the nearest endocrinology or cardiology clinic were calculated for each zip code centroid in ArcGIS Pro version 3.2 (Esri) using an origin-destination cost matrix to calculate the shortest driving route. Rural-urban commuting area codes based on 5-digit zip code were categorized into urban, large rural city or town, and small or isolated small rural town.^25^ Local area walkability was assessed using the Environmental Protection Agency’s National Walkability Index, a weighted sum of walkability indicators ranked nationally (range, 1-20 with higher scores indicating greater walkability).^26^ Public transit accessibility was defined using density per square mile of public transit stops using the Department of Transportation Bureau of Transportation Statistics National Transit Map.^27^ Cellular data and broadband internet access were assessed using measures from the 2021 American Community Survey indicating the percentage of residents who reported access to each.^28^ Federal Communications Commission Fixed Broadband Deployment Data was used to assess whether mean broadband download speed was greater than or equal to 25 Mpbs, because this speed is generally required for audio-visual communication.

### Sociodemographic Measures

Demographic characteristics, including age, gender, race (Asian, Black, White, and other, which includes Alaska Native, American Indian, Chamorro, Hawaiian, Samoan, Other Pacific Islander, declined, not specified, and unknown), and ethnicity (Hispanic, not Hispanic, and not specified) were collected from medical record data, based on patient self-report upon entry into the health system. Social Deprivation Index, a composite measure of local area deprivation linked to health outcomes ranging from 0 to 100, with higher values indicating greater deprivation,^29^ was assigned to each patient using the zip code Tabulation Area to zip code crosswalk by the Health Resources and Services Administration. The Elixhauser comorbidity index, a measure of multimorbidity that ranges from −19 to 89, with higher scores indicating greater comorbidity and has been linked to adverse outcomes for both diabetes and ASCVD,^30,31^ was calculated on the basis of comorbidities documented at least twice in outpatient encounters from 2016 to 2018.

### Statistical Analysis

We summarized baseline patient characteristics using frequencies for categorical variables and means for continuous variables as appropriate. Because more than 25% of patients resided in zip codes with a mean of 0 transit stops per square mile, public transit stop density was categorized into quartiles. Baseline characteristics were compared among those who did vs did not receive endocrinology or cardiology care over the entire study period using χ^2^ tests for categorical variables and Kruskal-Wallis tests for continuous variables.

The primary outcome variables were whether each patient had at least 1 visit with either cardiology or endocrinology during the pretelemedicine period and separately during the posttelemedicine period. We used multivariable logistic regression to model the association of geographic and sociodemographic factors with the use of each type of specialty care. We stratified analyses on the basis of the pretelemedicine and posttelemedicine time periods to examine differences in the association of key patient-level variables with specialty care utilization before and after widespread telemedicine availability. We then fit an interaction-based multilevel logistic regression model to formally test for differences in the association of these variables and care utilization between pretelemedicine and posttelemedicine periods. Specifically, we included interactions between period (pretelemedicine vs posttelemedicine) and all independent variables from stratified models. A random effect for patient was included to account for 2 repeated measures per patient in mixed models. Drive distance to the closest cardiology or endocrinology clinic concordant with the primary outcome was used in each model. Variables were checked for collinearity: broadband access, broadband speed over 25 megabits per second, and cellular data access were colinear, as were driving time and distance. Given the limited variability in broadband access and speed across zip codes and the importance of cellular data in access to telemedicine outside of the home, broadband access and speed were removed from models. Driving distance was chosen over time for ease of interpretation and given greater variation within the cohort. Participants for whom zip code could not be mapped were excluded from models (86 participants); race and ethnicity were the only sociodemographic variables with missing data, and these participants were included in all models by coding a level for missing. All analyses assumed a type I error rate of α = .05 with no adjustments made for multiplicity and were performed using SAS statistical software version 9.4 (SAS Institute).

## Results

### Cohort Characteristics

**Figure 1.**
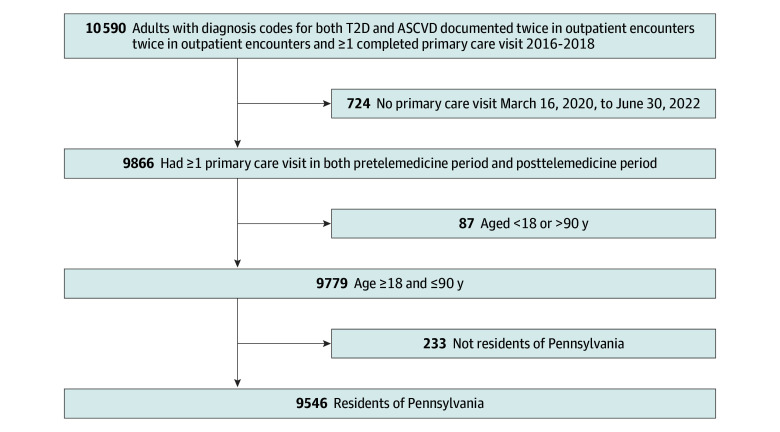
Cohort Derivation ASCVD indicates atherosclerotic cardiovascular disease; T2D, type 2 diabetes.

**Figure 2.**
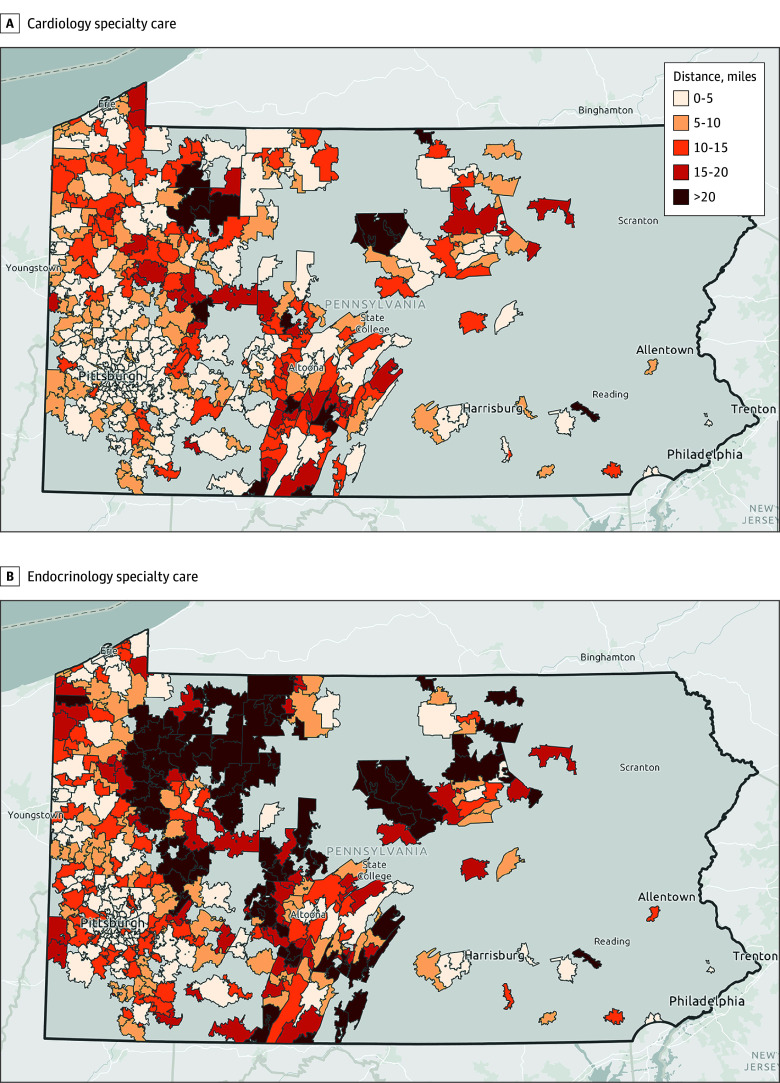
Shortest Driving Distance in Miles From Population-Weighted Zip Code Centroid to Care Centers Maps show driving distance to cardiology specialty care (A; range, 0.03-42.2 miles) and endocrinology specialty care (B; range, 0.02-55.3 miles). Maps were created by E.K.

There were 9546 patients in the final cohort (mean [SD] age, 68.5 [10.0] years; 5854 male [61%]; 82 Asian [1%]; 930 Black [10%]; 8451 White [89%]; 9149 non-Hispanic or Latino [96%]; 7877 urban [83%]) (Figure 1 and Table 1). Across the entire study period, 5578 patients received cardiology care and 1747 received endocrinology care. More cardiology clinic locations (742 locations, with an estimated 433 cardiology practitioners) were identified than endocrinology (280 locations, with an estimated 186 endocrinology practitioners) across the state (eFigure in Supplement 1). Patients lived in 514 zip codes (mean [SD], 18.6 [33.9] patients per zip code; median [IQR], 4.0 [1.0-19.0] patients per zip code). The drive distance from population-weighted zip code centroid to a cardiology clinic was shorter (mean [SD], 3.7 [4.2] miles; median [IQR], 2.4 [1.0-4.6] miles) compared with endocrinology clinics (mean [SD], 5.5 [6.5] miles; median [IQR], 3.1 [1.4-7.0] miles) (Figure 2). In unadjusted analyses, adults who received endocrinology care were slightly younger, more likely to be Black, live in an urban area, have higher neighborhood socioeconomic status and walkability, and to live within a shorter driving distance to the nearest endocrinology clinic compared with those who did not (Table 1). People who received cardiology care were more likely to live in urban areas, had slightly higher local area socioeconomic status and walkability index, and were more likely to have higher Elixhauser comorbidity scores than those who did not (Table 1).

**Table 1.** Characteristics of Patients Receiving vs Not Receiving Specialty Care

Characteristic	Total (N = 9546), No. (%)	Endocrinology care			Cardiology care		
		Yes (n = 1747), No. (%)	No (n = 7779), No. (%)	*P* value	Yes (n = 5578), No. (%)	No (n = 3968), No. (%)	*P* value
Age, mean (SD), y	68.5 (10.0)	65.3 (9.9)	69.2 (9.9)	<.001^a^	68.5 (9.8)	68.4 (10.3)	.59^a^
Gender							
Female	3692 (39)	745 (43)	2947 (38)	<.001^b^	2106 (38)	1586 (40)	.03^b^
Male	5854 (61)	1002 (57)	4852 (62)		3472 (62)	2382 (60)	
Race							
Asian	82 (1)	25 (1)	57 (1)	<.001^b^	54 (1)	28 (1)	.08^b^
Black	939 (10)	227 (13)	712 (9)		517 (9)	422 (11)	
White	8451 (89)	1482 (85)	6969 (89)		4962 (89)	3489 (88)	
Other or missing^c^	74 (1)	13 (1)	61 (1)		45 (1)	29 (1)	
Ethnicity							
Hispanic	49 (1)	15 (1)	34 (<1)	.08^b^	30 (1)	19 (<1)	.46^b^
Not Hispanic	9149 (96)	1667 (95)	7482 (96)		5334 (96)	3815 (96)	
Not specified	348 (4)	65 (4)	283 (4)		214 (4)	134 (3)	
Social Deprivation Index score, mean (SD)	40.1 (23.7)	41.5 (24.7)	39.8 (23.5)	.06^a^	39.7 (24.1)	40.6 (23.2)	.04^a^
Rural-urban commuting area							
Urban	7877 (83)	1532 (88)	6345 (81)	<.001^b^	4765 (85)	3112 (78)	<.001^b^
Large rural town	1008 (11)	134 (8)	874 (11)		500 (9)	508 (13)	
Small rural town	661 (7)	81 (5)	580 (7)		313 (6)	348 (9)	
Cellular data service, mean (SD), % of population^d^	62.8 (6.7)	63.1 (6.5)	62.7 (6.7)	.03^a^	63.1 (6.7)	62.2 (6.6)	<.001^a^
Elixhauser Comorbidity Index score							
<0	1840 (19)	421 (24)	1419 (18)	<.001^b^	1042 (19)	798 (20)	<.001^b^
0	1831 (19)	228 (13)	1603 (21)		855 (15)	976 (25)	
1-5	2789 (29)	492 (28)	2297 (29)		1594 (29)	1195 (30)	
6-13	2243 (23)	431 (25)	1812 (23)		1474 (26)	769 (19)	
≥14	843 (9)	175 (10)	668 (9)		613 (11)	230 (6)	
Mean Walkability Index, mean (SD)^d^	9.8 (3.2)	10.2 (3.3)	9.8 (3.2)	<.001^a^	10.0 (3.2)	9.6 (3.3)	<.001^a^
Transit stops density by quartile^d^							
First (zero)	4117 (44)	729 (42)	3388 (44)	<.001^b^	2393 (43)	1724 (44)	.73^b^
Second (<0.3)	619 (7)	113 (6)	506 (7)		363 (7)	256 (7)	
Third (0.3-13.7)	2361 (25)	381 (22)	1980 (26)		1367 (25)	994 (25)	
Fourth (13.8-119.2)	2363 (25)	517 (30)	1846 (24)		1403 (25)	960 (24)	
Distance to closest relevant specialty clinic, mean (SD), miles^d^	NA	4.6 (5.2)	5.7 (6.7)	<.001^a^	3.6 (4.2)	3.8 (4.3)	.11^a^

Abbreviation: NA, not applicable.

^a^ Calculated with Wilcoxon rank sum test.

^b^ Calculated with χ^2^ test.

^c^ Other category includes Alaska Native, American Indian, Chamorro, Hawaiian, Samoan, Other Pacific Islander, declined, not specified, and unknown.

^d^ Data are missing for 86 participants.

### Factors Associated With Receipt of Endocrinology Care Pretelemedicine vs Posttelemedicine

In the pretelemedicine period, 1321 patients received endocrinology care, whereas 1398 received care posttelemedicine. In multivariable logistic regression modeling for the pretelemedicine period, greater distance to endocrinology clinic was negatively associated with receipt of endocrinology care (adjusted odds ratio [aOR] per 10 miles, 0.74; 95% CI, 0.64-0.84), whereas higher neighborhood walkability was positively associated with care receipt (aOR per 10 points, 1.38; 95% CI, 1.00-1.91) (Table 2). With respect to sociodemographic variables, race was significantly associated with greater odds of receipt of endocrinology care (Black patients compared with White patients, aOR, 1.21; 95% CI, 0.99-1.47; Asian patients compared with White patients, aOR, 2.55; 95% CI, 1.53-4.25; patients with other or missing race compared with White patients, aOR, 1.30; 95% CI, 0.70-2.40) (overall *P* = .003). Older age (aOR per 10 years, 0.70; 95% CI, 0.66-0.75), male gender (aOR for male vs female, 0.82; 95% CI, 0.72-0.92), and lower neighborhood-level socioeconomic status (aOR per 10 points of Social Deprivation Index, 0.95; 95% CI, 0.91-0.99) were associated with lower odds of receiving endocrinology care. Increasing comorbidity index was also associated with receipt of endocrinology care (aOR for ≥14 vs <0, 1.27; 95% CI, 1.02-1.59). In the posttelemedicine period, neighborhood walkability, neighborhood-level socioeconomic status, and race were not significantly associated with care receipt, whereas age, gender, and comorbidity index had similar associations as in the pretelemedicine period. The percentage of residents with cellular data was not associated with endocrinology care use in either the pretelemedicine or posttelemedicine period. In mixed models comparing the associations of factors with receipt of care in the pretelemedicine vs posttelemedicine periods, the aORs for receipt of endocrinology care increased for distance to endocrinology clinic, race, and comorbidity index in the posttelemedicine period compared with the pretelemedicine period, but the aOR for older age decreased in the posttelemedicine period.

**Table 2.** Factors Associated With Receipt of Endocrinology Care Pretelemedicine vs Posttelemedicine in Multivariable Models

Patient-level factor	Pretelemedicine (n = 1321)		Posttelemedicine (n = 1398)		Pretelemedicine vs posttelemedicine *P* value^b^
	aOR (95% CI)^a^	*P* value	aOR (95% CI)^a^	*P* value	
Distance to endocrinology clinic (per 10 miles)	0.74 (0.64-0.84)	<.001	0.82 (0.73-0.93)	.002	.047
Percentage of population with cellular data (per 10%)	0.97 (0.84-1.11)	.62	1.03 (0.90-1.19)	.64	.48
Transit stop density					
Quartile 2 vs quartile 1	0.97 (0.75-1.24)	.001	1.16 (0.92-1.47)	.003	.08
Quartile 3 vs quartile 1	0.72 (0.61-0.85)		0.77 (0.66-0.91)		
Quartile 4 vs quartile 1	0.92 (0.75-1.12)		0.93 (0.77-1.14)		
Mean Walkability Index (per 10 points)	1.38 (1.00-1.91)	.047	1.33 (0.97-1.82)	.08	.63
Age (per 10 y)	0.70 (0.66-0.75)	<.001	0.67 (0.63-0.71)	<.001	.02
Gender (male vs female)	0.82 (0.72-0.92)	.001	0.83 (0.74-0.94)	.002	.56
Race					
Black compared with White	1.21 (0.99-1.47)	.003	0.93 (0.76-1.14)	.09	<.001
Asian compared with White	2.55 (1.53-4.25)		1.85 (1.09-3.16)		
Other or missing compared with White^c^	1.30 (0.70-2.40)		0.71 (0.35-1.44)		
Ethnicity					
Hispanic or Latino compared with not	1.46 (0.71-2.97)	.32	1.77 (0.91-3.45)	.25	.08
Missing compared with not Hispanic or Latino	0.84 (0.60-1.16)		1.02 (0.76-1.39)		
Social Deprivation Index (per 10 points)	0.95 (0.91-0.99)	.02	0.98 (0.94-1.02)	.23	.21
Elixhauser Comorbidity Index score					
0 vs <0	0.52 (0.42-0.63)	<.001	0.57 (0.46-0.69)	<.001	.02
1-5 vs <0	0.85 (0.72-1.01)		0.95 (0.81-1.12)		
6-13 vs <0	0.98 (0.82-1.17)		1.06 (0.90-1.26)		
≥14 vs <0	1.27 (1.02-1.59)		1.13 (0.90-1.42)		

Abbreviation: aOR, adjusted odds ratio.

^a^ Multivariable logistic regression models including all above variables.

^b^ Interaction-based multilevel logistic regression model tested for differences in associations between infrastructure variables and care receipt between pretelemedicine and posttelemedicine periods.

^c^ Other category includes Alaska Native, American Indian, Chamorro, Hawaiian, Samoan, Other Pacific Islander, declined, not specified, and unknown.

### Factors Associated With Receipt of Cardiology Care Pretelemedicine vs Posttelemedicine

In the pretelemedicine period, 4799 patients received cardiology care, whereas 4601 had at least 1 visit in the posttelemedicine period. In multivariable logistic regression models for the pretelemedicine period, greater local area cellular data access (aOR per 10%, 1.19; 95% CI, 1.09-1.31) and walkability index (aOR per 10 points, 1.60; 95% CI, 1.27-2.01) were associated with receipt of cardiology care (Table 3). Drive distance to cardiology clinic was not significantly associated with receipt of cardiology care pretelemedicine. Black patients had lower odds of care receipt compared with White patients (aOR, 0.71; 95% CI, 0.60-0.82), and Asian patients had greater odds compared with White patients (aOR, 1.14; 95% CI, 0.72-1.79) (overall *P* < .001), whereas age, gender, and neighborhood socioeconomic status were not associated. As seen with endocrinology care, higher comorbidity index (aOR for ≥14 vs <0, 2.19; 95% CI, 1.82-2.61) was positively associated with receipt of cardiology care pretelemedicine. In the posttelemedicine period, greater cellular data access and neighborhood walkability were still positively associated with care receipt, and associations of demographic factors with cardiology care were similar to the pretelemedicine period. In mixed models comparing the pretelemedicine vs posttelemedicine periods, the aOR for greater distance to cardiology clinic increased slightly in the posttelemedicine period, whereas that for comorbidity index decreased.

**Table 3.** Factors Associated With Receipt of Cardiology Care Pretelemedicine vs Posttelemedicine in Multivariable Models

Patient-level factor	Pretelemedicine (n = 4799)		Posttelemedicine (n = 4601)		Pretelemedicine vs posttelemedicine *P* value^b^
	aOR (95% CI)^a^	*P* value	aOR (95% CI)^a^	*P* value	
Distance to cardiology clinic (per 10 miles)	0.99 (0.88-1.11)	.84	1.10 (0.98-1.23)	.11	.02
Percentage of population with cellular data (per 10%)	1.19 (1.09-1.31)	<.001	1.11 (1.01-1.22)	.03	.23
Transit stop density					
Quartile 2 vs quartile 1	1.07 (0.90-1.27)	.02	0.98 (0.83-1.17)	.07	.04
Quartile 3 vs quartile 1	0.85 (0.76-0.95)		0.90 (0.81-1.01)		
Quartile 4 vs quartile 1	0.90 (0.78-1.04)		0.83 (0.72-0.96)		
Mean Walkability Index (per 10 points)	1.60 (1.27-2.01)	<.001	1.64 (1.30-2.06)	<.001	.86
Age (per 10 y)	0.98 (0.94-1.03)	.42	0.98 (0.94-1.02)	.29	.8
Gender (male vs female)	1.08 (0.99-1.18)	.07	1.14 (1.05-1.24)	.003	.26
Race					
Black compared with White	0.71 (0.60-0.82)	<.0	0.74 (0.64-0.87)	.001	.91
Asian compared with White	1.14 (0.72-1.79)		1.21 (0.77-1.90)		
Other or missing compared with White^c^	0.96 (0.60-1.55)		0.91 (0.57-1.46)		
Ethnicity					
Hispanic or Latino compared with not	0.92 (0.52-1.64)	.49	1.10 (0.62-1.94)	.67	.94
Missing vs not Hispanic compared with Latino	1.14 (0.91-1.42)		1.10 (0.88-1.37)		
Social Deprivation Index (per 10 points)	0.98 (0.95-1.01)	.13	0.98 (0.95-1.00)	.09	.74
Elixhauser Comorbidity Index					
0 vs <0	0.68 (0.59-0.77)	<.001	0.77 (0.67-0.88)	<.001	<.001
1-5 vs <0	1.01 (0.89-1.13)		1.10 (0.97-1.24)		
6-13 vs <0	1.53 (1.34-1.74)		1.46 (1.28-1.65)		
≥14 vs <0	2.19 (1.82-2.61)		2.01 (1.69-2.39)		

Abbreviation: aOR, adjusted odds ratio.

^a^ Multivariable logistic regression models including all above variables.

^b^ Interaction-based multilevel logistic regression model tested for differences in associations between infrastructure variables and care receipt between pretelemedicine and posttelemedicine periods.

^c^ Other category includes Alaska Native, American Indian, Chamorro, Hawaiian, Samoan, Other Pacific Islander, declined, not specified, and unknown.

## Discussion

In this retrospective cohort study, we identified geographic and sociodemographic factors associated with receipt of metabolic disease specialty care among adults with T2D and ASCVD in a large, geographically diverse health system. Increasing distance to clinic and age were negatively associated with receipt of endocrinology care, whereas race was associated with differential receipt of cardiology care when adjusted for other factors that may impact care access. In addition, greater comorbidity burden was significantly associated with both endocrinology and cardiology care receipt.

Over 3 times as many patients received cardiology than endocrinology care, with more cardiology clinic locations resulting in significantly shorter mean distance to the nearest clinic. These differences in availability and access to in-person care could explain some differences in associations between patient-level factors and care receipt between the 2 specialties. For endocrinology care in the pretelemedicine period, greater distance to clinic was significantly associated with lower odds of care, and this association decreased after telemedicine expansion. For cardiology care, distance to clinic was not associated with receipt of care in either the pretelemedicine or posttelemedicine period, whereas race, comorbidity burden, and neighborhood characteristics, such as cellular data access and walkability, were. With fewer clinic locations and high utilization of telemedicine,^24^ geographic barriers may have had greater association with access to endocrinology care pretelemedicine, with more improvement in access after telemedicine expansion. On the other hand, cardiology care may be more accessible in person but have less uptake of telemedicine,^24^ resulting in greater impact of nongeographic factors on access to care.

Although the paucity of endocrinology and cardiology care in rural areas of the US is well documented,^18,32^ to our knowledge, this is the first study to evaluate the association of geographic barriers and other patient-level factors with receipt of this care. With an absence of national guidelines on which patients should receive specialty care for T2D and ASCVD, it is crucial to understand how these factors currently impact utilization of this limited care resource. For both specialties, patients with more comorbidities were more likely to receive care, suggesting that clinical complexity appropriately influences care receipt. Other clinical factors not captured in this analysis, such as disease complications and severity, likely also impact receipt of specialty care. However, nonclinical factors, including distance to clinic and race, were also significantly associated with receipt of endocrinology and cardiology care, respectively. Higher cardiovascular disease risk, higher mortality, and lower care quality are well documented for underresourced populations with diabetes.^5,8,10,13,33^ Thus, ensuring that clinical factors are the primary drivers of specialty care receipt by facilitating equitable access to care may be one of many critical strategies to address these disparities.

This study also adds new evidence on the potential association of telemedicine with access to metabolic disease specialty care. Telemedicine alleviates transportation barriers, which are more likely to affect underresourced populations.^20^ However, the association of telemedicine with access to care for patients in rural areas is not clear: rural populations may be more likely to utilize telemedicine,^34^ but there is also concern that telemedicine may exacerbate disparities in access to care in rural areas with limited broadband internet access.^35,36^ In this study, we found that telemedicine may have facilitated access for patients facing geographic barriers to endocrinology care. Therefore, ongoing insurance coverage and use of telemedicine are crucial to sustain more-equitable access for patients who live at greater distances from endocrinology clinics. In addition, efforts to ensure that the quality of T2D care delivered via telemedicine is comparable to that of in-person care are needed to support translation of increased access to improved clinical outcomes for these populations. We also found that older people were less likely to receive endocrinology care in the posttelemedicine period, aligning with prior work.^37^ Telemedicine also did not alter the negative association of Black race with receipt of cardiology care. Thus, additional strategies are needed to understand and address the factors underlying observed disparities in receipt of metabolic disease specialty care by age and race.

### Limitations

Although our findings add novel information on the association of geographic and sociodemographic factors with specialty care receipt, this study has several limitations. First, the observational nature of this study limits inferences about the causal relationships among these factors of interest and specialty care receipt. Second, the use of 5-digit zip codes as the smallest geographic unit may limit specificity of geographic variables. Third, this work was conducted in a large, but single, health system and was limited to a population who regularly engaged with primary care, so results may not be generalizable to other settings or patients. Fourth, additional patient-level barriers that likely impact specialty care receipt, such as insurance type and primary care practitioner referral patterns, were not available in this dataset and, therefore, were not assessed. Fifth, although comorbidity burden was associated with receipt of both types of specialty care, additional clinical factors that may impact specialty care use, such as hemoglobin A_1c_ level, complications, and type and severity of ASCVD, were not evaluated in this study. Assessing the association of these factors with receipt of specialty care across multiple health systems will be an important next step in continuing to understand how a patient’s place of residence, sociodemographic, and clinical characteristics impact receipt of metabolic disease specialty care.

## Conclusions

In this cohort study of adults with T2D and ASCVD, geographic and sociodemographic factors associated with receipt of specialty care differed between endocrinology and cardiology. Introduction of telemedicine reduced the impact of geographic barriers to endocrinology care, but was also associated with lower odds of care receipt for older patients. Widespread use of telemedicine did not significantly modify disparities in utilization of cardiology care by race. As underresourced populations face worse clinical outcomes and multiple barriers to care, continued evaluation of factors that affect care access and utilization across diverse care settings can inform approaches to promote equitable utilization of metabolic disease specialty care and improve clinical outcomes for adults with T2D and ASCVD.

## Notes

Supplement 1. **eFigure.** Cardiology and endocrinology specialty clinic locations with cohort patient count in 5-digit ZIP code

Supplement 2.
Data Sharing Statement
