# Supplementary material for: Geographic and Sociodemographic Factors and Receipt of Metabolic Disease Specialty Care
Source: JAMA Netw Open. 2025 May 20;8(5):e2511559. doi: 10.1001/jamanetworkopen.2025.11559 (PMC12093186; doi:10.1001/jamanetworkopen.2025.11559)
Supplement: Supplement 1. — eFigure. Cardiology and endocrinology specialty clinic locations with cohort patient count in 5-digit ZIP code [file jamanetwopen-e2511559-s001.pdf]

Supplemental Online Content

Zupa MF, Rothenberger SD, Bauer JG, et al. Geographic and sociodemographic factors and receipt of metabolic disease specialty care. *JAMA Netw Open*. 2025;8(5):e2511559. doi:10.1001/jamanetworkopen.2025.11559

eFigure. Cardiology and endocrinology specialty clinic locations with cohort patient count in 5-digit ZIP code

This supplemental material has been provided by the authors to give readers additional information about their work.

© 2025 Zupa MF et al. *JAMA Network Open*.

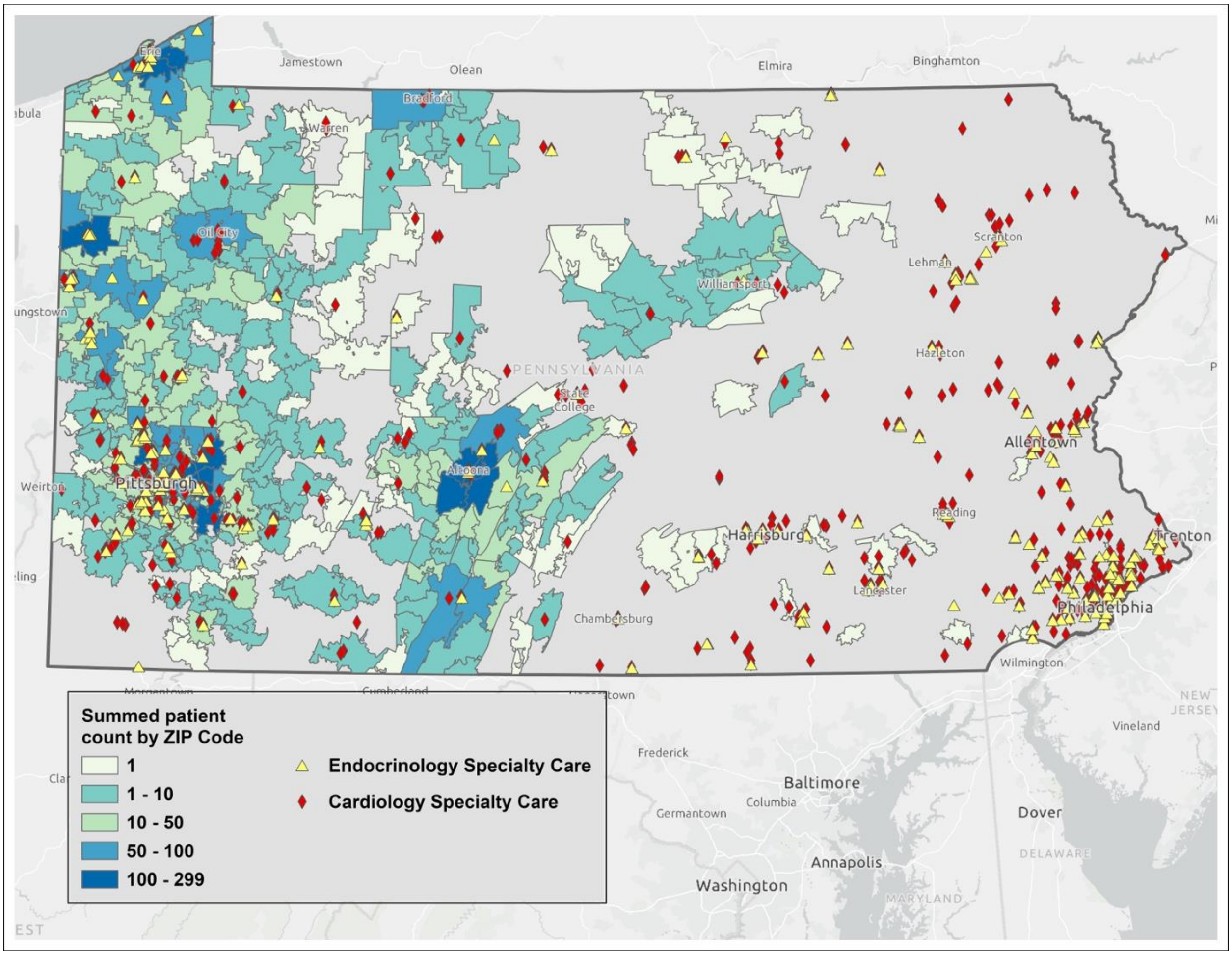

eFigure: Cardiology and endocrinology specialty clinic locations with cohort patient count in 5-digit ZIP code

© 2025 Zupa MF et al. *JAMA Network Open*.
