# Supplementary material for: Geographic and Sociodemographic Factors and Receipt of Metabolic Disease Specialty Care
Source: JAMA Netw Open. 2025 May 20;8(5):e2511559. doi: 10.1001/jamanetworkopen.2025.11559 (PMC12093186; doi:10.1001/jamanetworkopen.2025.11559)
Supplement: Supplement 2. — Data Sharing Statement [file jamanetwopen-e2511559-s002.pdf]

Data Sharing Statement

Zupa. Geographic and Sociodemographic Factors and Receipt of Metabolic Disease Specialty Care. *JAMA Netw Open*. Published May 20, 2025. doi:10.1001/jamanetworkopen.2025.11559

Data
Data available: No

Additional Information
Explanation for why data not available: The data used in this study include potentially identifying patient information including dates and zip codes, and thus are not available in their entirety. A limited dataset is available from the authors upon reasonable request and with permission of relevant institutional review boards.
